# Unsupervised learning of Swiss population spatial distribution

**DOI:** 10.1371/journal.pone.0246529

**Published:** 2021-02-11

**Authors:** Mikhail Kanevski

**Affiliations:** Institute of Earth Surface Dynamics, University of Lausanne, Lausanne, Switzerland; University of Essex, UNITED KINGDOM

## Abstract

The paper deals with the analysis of spatial distribution of Swiss population using fractal concepts and unsupervised learning algorithms. The research methodology is based on the development of a high dimensional feature space by calculating local growth curves, widely used in fractal dimension estimation and on the application of clustering algorithms in order to reveal the patterns of spatial population distribution. The notion “unsupervised” also means, that only some general criteria—density, dimensionality, homogeneity, are used to construct an input feature space, without adding any supervised/expert knowledge. The approach is very powerful and provides a comprehensive local information about density and homogeneity/fractality of spatially distributed point patterns.

## Introduction

The spatial distribution of the population depends on many environmental, social, and economic factors. Its analysis is an interesting and challenging scientific task with many practical applications. Depending on available data, very often the problem can be reformulated as a spatial point process (SPP). There exists a comprehensive literature devoted to the study of SPP, see for example, [1–7]. One of the most fundamental questions concerns spatial data clustering, which can be studied using different topological, statistical and fractal measures [8]. SPP and fractal theory, [9–17], are very popular in characterizing complex natural and social phenomena and data, especially when they are highly variable at many spatial and temporal scales.

One of very important and intensively studied topic in these fields deals with the analysis and modelling of spatio-temporal population distribution and evolution of cities [18–21]. Closely related questions concern the Zipf’s law, [22], which characterizes global distribution properties of data.

Recently, a new city clustering algorithm was proposed and successfully applied for clustering/aggregation/segmentation of the local information on population distribution into connected zones which can be attributed to the cities [23]. It was successfully applied to objectively define the cities and their distributions in the world.

In the present research a spatial distribution of the population in Switzerland is studied. The detailed description of the data and their first analysis using global multi-fractal approach was already carried out in [24]. The current paper concentrates on local analysis of population distribution using fractal concepts of dimension and scaling and clustering algorithms.

The data considered are of good quality and have very high spatial resolution (gridded data/cells: [100m x 100m]). In general, Switzerland can be considered as being composed of three geomorphological regions—the Swiss Alps, the Plateau and Jura, influencing the distribution of population, see details in [24]. Below, the same data are studied via representing them in a high dimensional feature space where unsupervised learning algorithms are applied to reveal patterns of population local distribution. Local properties of spatial data previously were also studied in different fields. Let us mention the following papers [7, 25, 26], which partly stimulated the present research.

The research follows a coherent methodology which consists of several important steps: advanced exploratory data analysis, including generation of simulated data in a validity domain, embedding of original data into a feature space composed of local growth curves, study of the clusterability (clustering tendency), selection and calibration of the unsupervised learning algorithms, understanding and qualitative interpretability of the results. This methodology forms the major part of the contribution to the novelty of the research.

The manuscript is organised as follows. First, some selected results on exploratory data analysis (EDA) are presented and visualised. Then, the construction of a high dimensional feature space is explained and explored. In the following section, different algorithms of clustering are applied to study patterns of spatial distribution of population. Finally, the results are discussed and future research topics are briefly mentioned. The bibliography includes relevant references.

## Data description

The original data were provided by the Swiss Federal Office of Statistics (https://www.bfs.admin.ch/bfs/en/home.html) and obtained via the access at the University of Lausanne (www.unil.ch). Original census data of 2010 were gridded with a resolution of [100*m* × 100*m*].

Before going to the details of the study, let us remind a concept of validity domain which was introduced and widely used to characterise environmental monitoring networks in geostatistical modelling and analysis of spatial point processes in complex regions [8, 16, 27].

Real data are almost always clustered due to some “natural clustering”. Real phenomena are not “living” in a pure mathematical space. For example, if we study forest fires, they should be considered in a forest, which can be highly clustered itself. In this case, the forest region is considered as a validity domain (VD), i.e. a region where the data are collected and the phenomena is studied. Even if we generate a completely random data set in the forest, corresponding pattern will demonstrate an important spatial clustering [8, 28]. Therefore, the answer on real data clustering is often hidden by non trivial structure of the validity domain.

One possibility to deal with this problem, is to apply different correction factors, taking into account, for example, the shape of the region under study [1–3]. Another possibility is to generate a CSR (complete spatial randomness) pattern in the validity domain and *compare* the results with the raw data. In general, the combination of both approaches may be the most complete and informative.

In the present research we will use VD concept, which is defined by the surface equal to the administrative Swiss border without the surface of the lakes. This data set is called “CHCSR” and is presented below as a simulated data set. This simple model of VD already presents some interesting properties useful for the study.

An important phase of any data science research deals with an exploratory (spatial) data analysis—E(S)DA, which can apply different (pre)processing and visualisation tools.

### Real data

According to the discussion above, populated cells are considered as spatial point data. Loosely speaking, it means that scales large than cell’s size (100 m) have to be considered.

**Fig 1:**
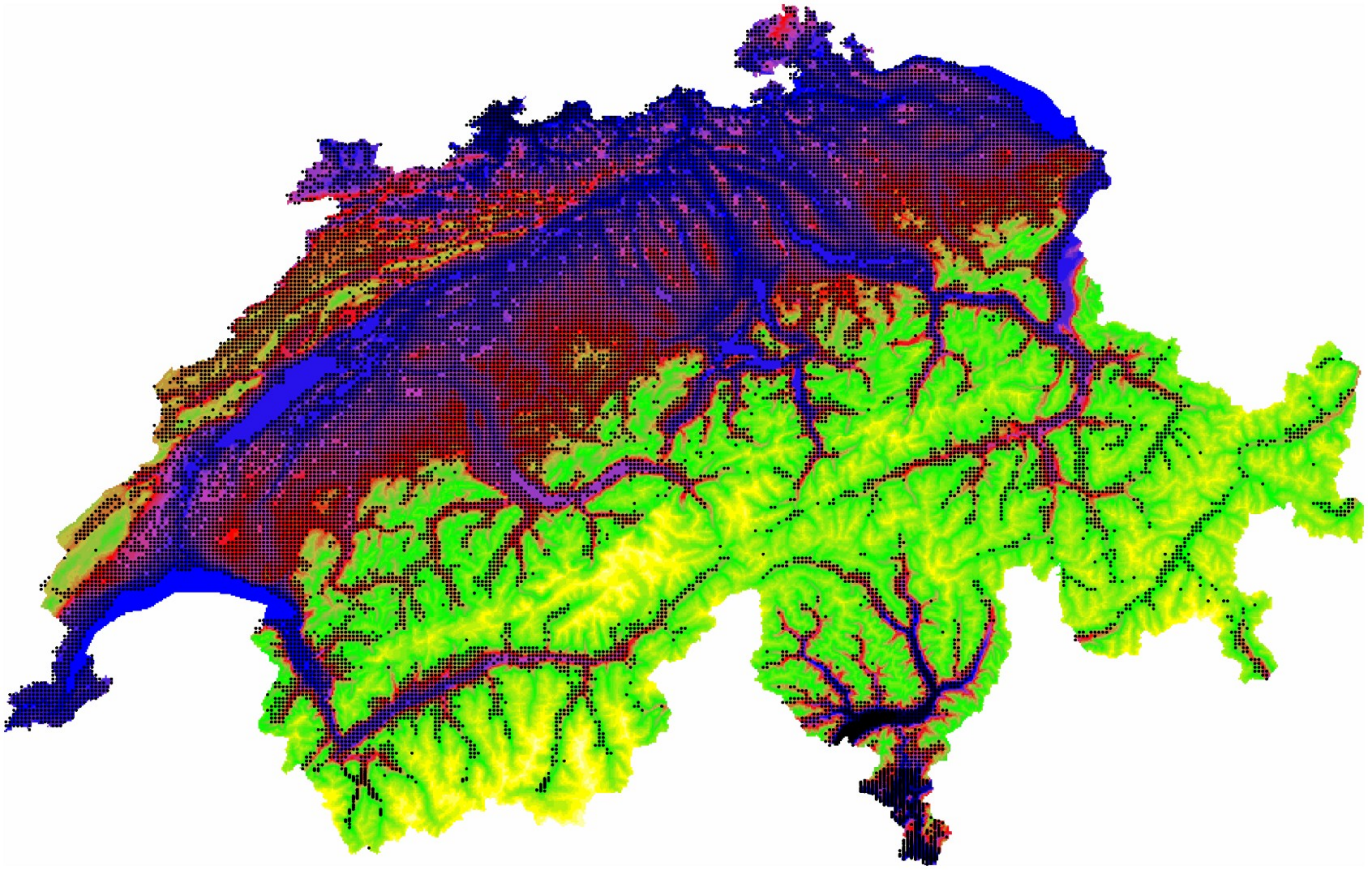
Visualisation of the spatial distribution of populated cells using digital elevation model (DEM).

The spatial distribution (data postplot) of the populated cells is presented in Fig 1. Let us note, that some figures are constructed using only a randomly selected part (sometimes only ∼ 10%) of data for the visualisation purposes.

Of course, data are highly spatially clustered. The clustering observed is a result of many interacting factors, including topography and geomorphology of the territory.

The number of populated cells equals approximately to 330000, with a wide variation (three orders of magnitude) of the number of inhabitants per cell. The spatial data are visualized using the Swiss projection and coordinates are measured in meters. In the following presentation mainly two geographical layers—administrative limits of the Swiss communes and the lakes (blue polygons), are used. Only this information was also used to define a model of validity domain.

### Simulated data

Let us present a simulated data set, called CHCSR (Swiss Complete Spatial Randomness pattern). It was generated with the same number of points like in the raw population data within the boundary of Switzerland with the exclusion of the internal lakes.

**Fig 2:**
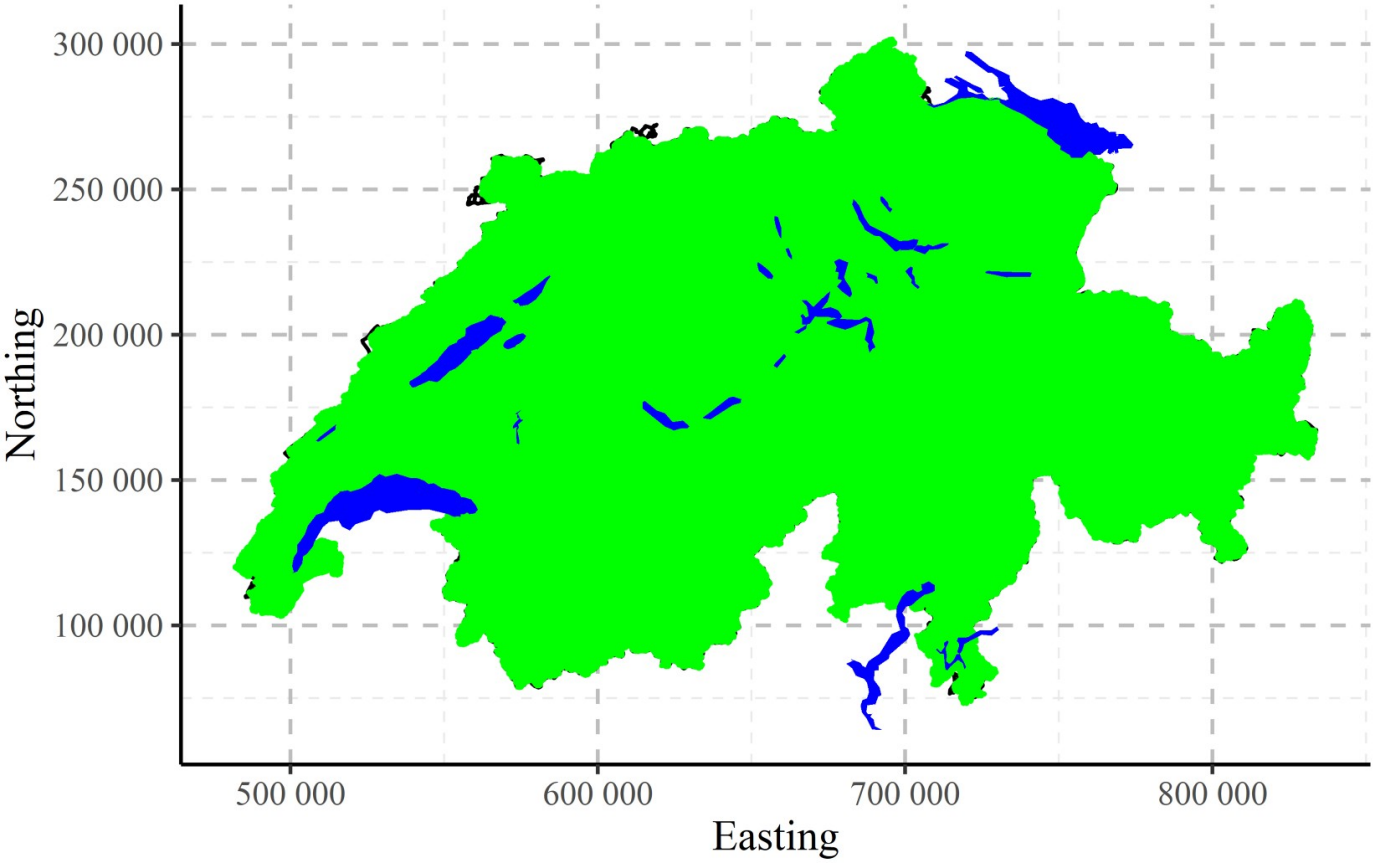
Spatial distribution of CHCSR pattern cells.

The postplot of the CHCSR pattern is shown in Fig 2. It has also some natural clustering, relating to the Swiss border and the spatial distribution of the lakes.

The considered two data sets are embedded into a high dimensional feature space, where unsupervised algorithms are applied, following the methodology described below.

## Methodology and methods

The generic methodology proposed in the research consists of several major phases:

- Exploratory (spatial) data analysis and visualization. Data cleaning and preprocessing.
- Spatial statistics and point processes analysis. Application of Morisita index.
- Local and global fractal analysis of real and simulated data. Box and sandbox counting methods.
- Analysis of clustering tendency. Selection of the number of clusters.
- Selection and application of unsupervised machine learning algorithms following the methodology of data clustering. Application of clustering methods: *k*–means, *k*–medoids (pam, CLARA) and self–organising (Kohonen) maps.
- Understanding and interpretation of the results.

All methods are applied to both real and simulated data. It helps in better understanding of data, algorithms and the results comparisons.

Different global clustering measures, useful to characterise data in a high dimensional space, are introduced as well. It is important, that global analysis completes the picture and provides useful information on intrinsic dimension and redundancy in data.

### Global analysis

There are many measures, developed in spatial statistics to study and quantify data clustering, see, for example, [1–3, 5, 6]. Loosely speaking, they can be classified as topological, statistical and fractal measures [8, 16]. Most of the analyses are carried out considering data set as a whole, ignoring local details by averaging them. Such approach is called a global analysis.

Below, three measures are selected to quantify global properties: Morisita index, box and sandbox counting estimations of the fractal dimension [13, 14, 16, 29].

### Fractal dimension

The literature, devoted to the theory and applications of fractal concepts in physics, biology, environmental sciences, social, economic, etc. domains, is extremely large and variable, see, for example, [9–11, 16, 17, 21, 25].

Box counting and sand box counting algorithms are standard techniques in estimation of fractal dimension (fDim).

Box counting method estimates fDim by covering the region of the study by a regular grid and counts how many cells are occupied at least by one point. Changing the number of grid cells (the resolution, *l*) we get a curve—*N*(*l*) ∼ *l*^−(*fb*)^, where *fb* is a fractal dimension estimated by box counting.

In case of sand box dimension estimation each point is a centre of the following calculation: we count how many other points fall into the “circle” (in the high dimensional space “hypersphere”) of the radius *R*_*i*_ [13, 14, 16]. After visiting all points with a given radius we average the number of points < *N*(*R*_*i*_) >. The curve $<N\left(R_{i}\right)>∼R_{i}^{fsb}$ defines the fractal dimension estimated via sand box approach. The computations are similar to the well known in spatial statistics Ripley’s *k*–function [1], or, in theory of dynamical systems, Grassberger–Procaccia correlation dimension [15].

**Fig 3:**
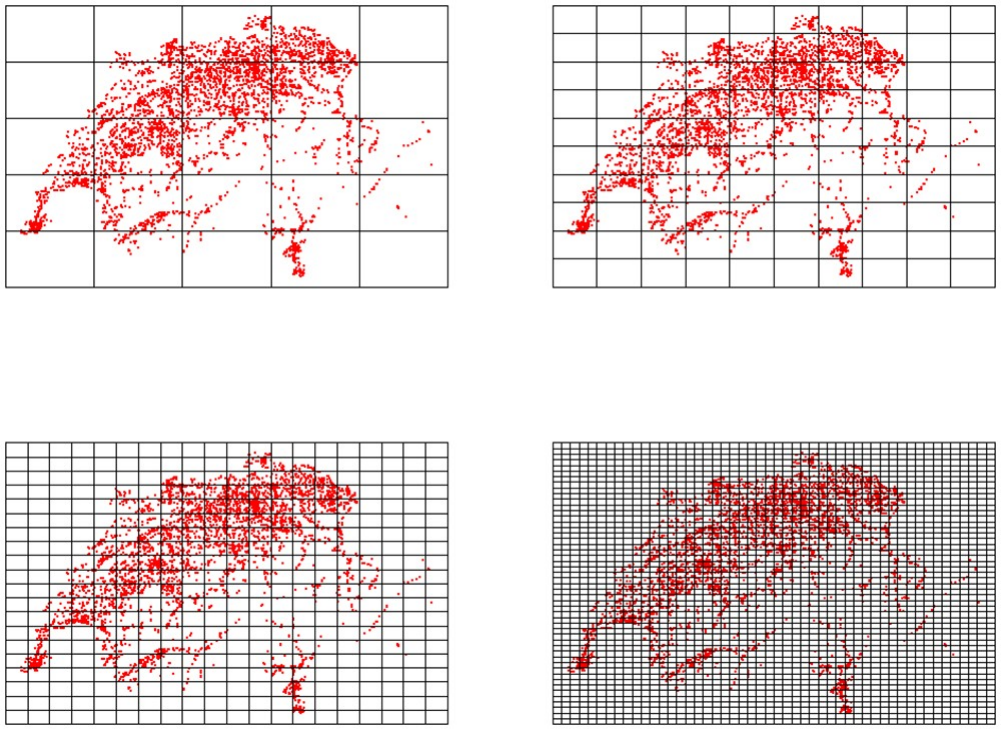
Principle of box counting estimation of the fractal dimension.

**Fig 4:**
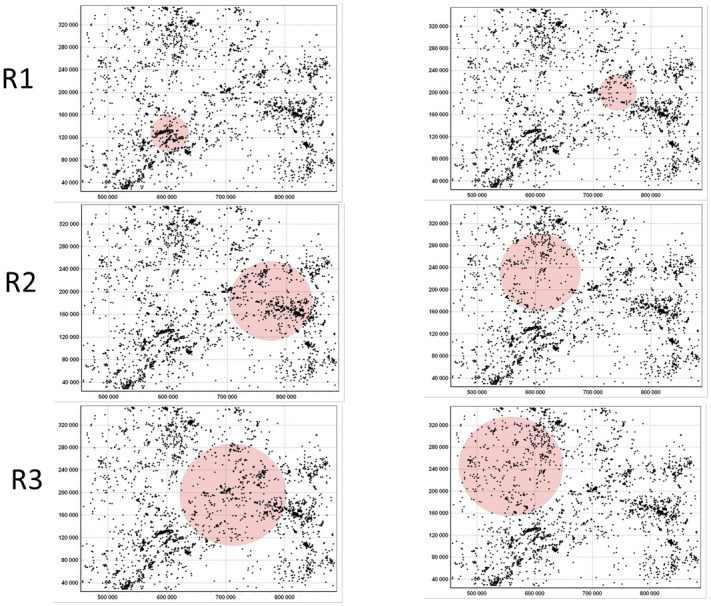
Principle of sand box counting estimation of the fractal dimension.

The principles of the box and sand box counting methods are illustrated in Figs 3 and 4.

**Fig 5:**
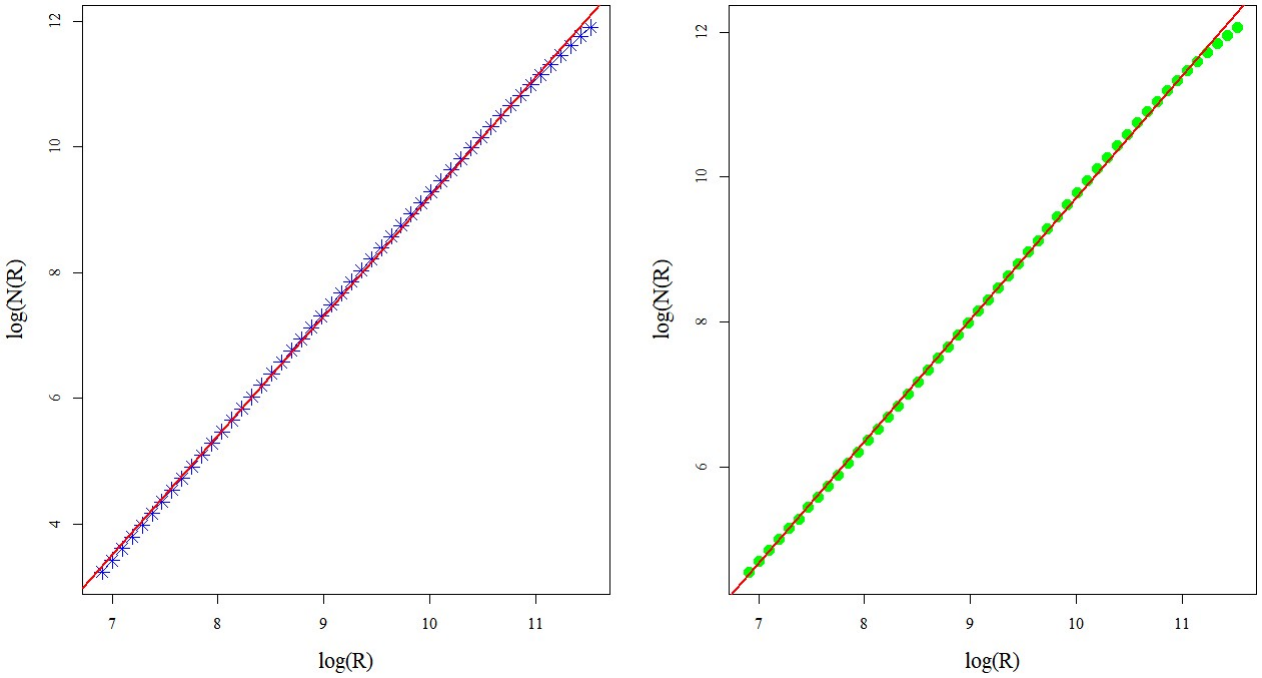
Sand box counting estimations of the CHCSR and Swiss population fractal dimensions.

Corresponding log-log curves, used to estimate sand box fractal dimensions for the CHCSR and real population data, are presented in Fig 5. The deviation of the fractal dimension for CHCSR (left, *fd*_*CHCSR*_ = 1.9) from *fd* = 2 (homogeneous pattern in 2*d*) is related to the validity domain. Fractal dimension for the real data *fd*_*CHpop*_ = 1.68 reflects the heterogeneity of the Swiss population distribution.

**Fig 6:**
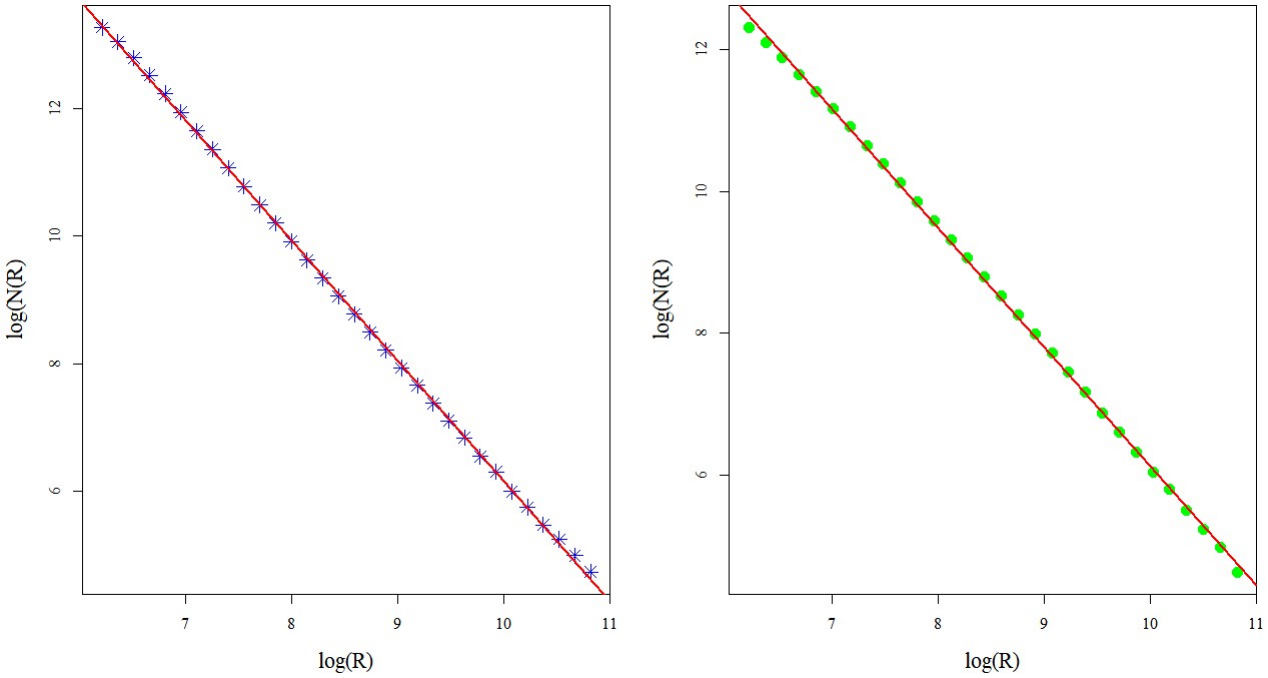
Box counting estimations of the CHCSR and Swiss population fractal dimensions.

The corresponding dimensions, estimated by box counting algorithm, are very close to the sand box estimations: for CHCSR pattern, *Fbox*_*CHCSR*_ = 1.88 and *Fbox*_*CHpop*_ = 1.68. Corresponding log-log dependencies for CHCSR (left) and Swiss population data (right) are shown in Fig 6.

### Morisita index

Morisita index (MI) was introduced in the fifties of the 20^th^ century by M. Morisita [29]. The classical version is a two-points index: *m* = 2. Later, it was generalised, by introducing multipoint Morisita curves [30] and, finally, a complete multipoint Morisita index (m-Morisita) was introduced and described with the case studies in [27].

Recently, it was shown how the index is connected to the fractality and intrinsic dimension estimation of data, which opened a way to the feature selection problems in machine learning [31, 32].

The m-Morisita index is calculated according to the following formula:

$$\begin{array}{c} I_{\delta }=Q^{m-1}\frac{\sum_{i=1}^{Q}n_{i}\left(n_{i}-1\right)\left(n_{i}-2\right)\cdots \left(n_{i}-m+1\right)}{\left(N(N-1)(N-2)\cdots )(N-m+1\right)}, \end{array}$$ (1)

where *n*_*i*_ is the number of points in the cell, *m*—the degree of the index, *Q*—total number of cells.

The calculation of index is very simple: the region of the study is covered by a regular grid with *Q* cells and the number of data points in each cell, *n*_*i*_, is counting. Then, we change the number of cells, or spatial resolution, and continue the procedure.

It is quite similar to the box method presented above: just instead of counting the cells, occupied with one or more points, Morisita is counting the cells with two or more points (two-point Morisita index).

Morisita index can be efficiently used to discriminate between clustered, regular (structured) and homogeneous (random) patterns [16].

In the present research the *R*—*package* “IDmining”, which can be found on CRAN (https://cran.r-project.org/), was applied to perform all analysis corresponding to the Morisita index.

### Local growth curves

The principle of local growth curves (LGC) calculation is explained in detail in the previous section, where a sand box method was presenting. In order to estimate global fractal dimension, the curves *N*(*x*, *y*, *R*_*i*_) were averaged over all data points (*x*, *y*) for each radius *R*_*i*_. Then, the *log*—*log* curve was used to estimate the fractal dimension.

Local—at each measurement point (*x*, *y*), growth curves give very reach information about the pattern under study at different spatial resolution. In the following local analysis the LGC information is not averaging over data points. The slope of the curve at each spatial point is (loosely) interpreted as a local fractal dimension (local *fDim*). In general, the local fractal dimension can be higher than the dimension of embedding Euclidean space.

In the present paper local growth curves of dimension *d* = 25 (number of radii used, *R*_*i*_, *i* = 1 …, *d*) were estimated in the range of [300 m–10000 m]. The selection of this range is not a trivial question and depends on data and type of clustering. This range was empirically selected after several trials, taking into account the topology of points distributions and global measures. In fact, if the whole curve as a coherent object is considered, it is not critical.

**Fig 7:**
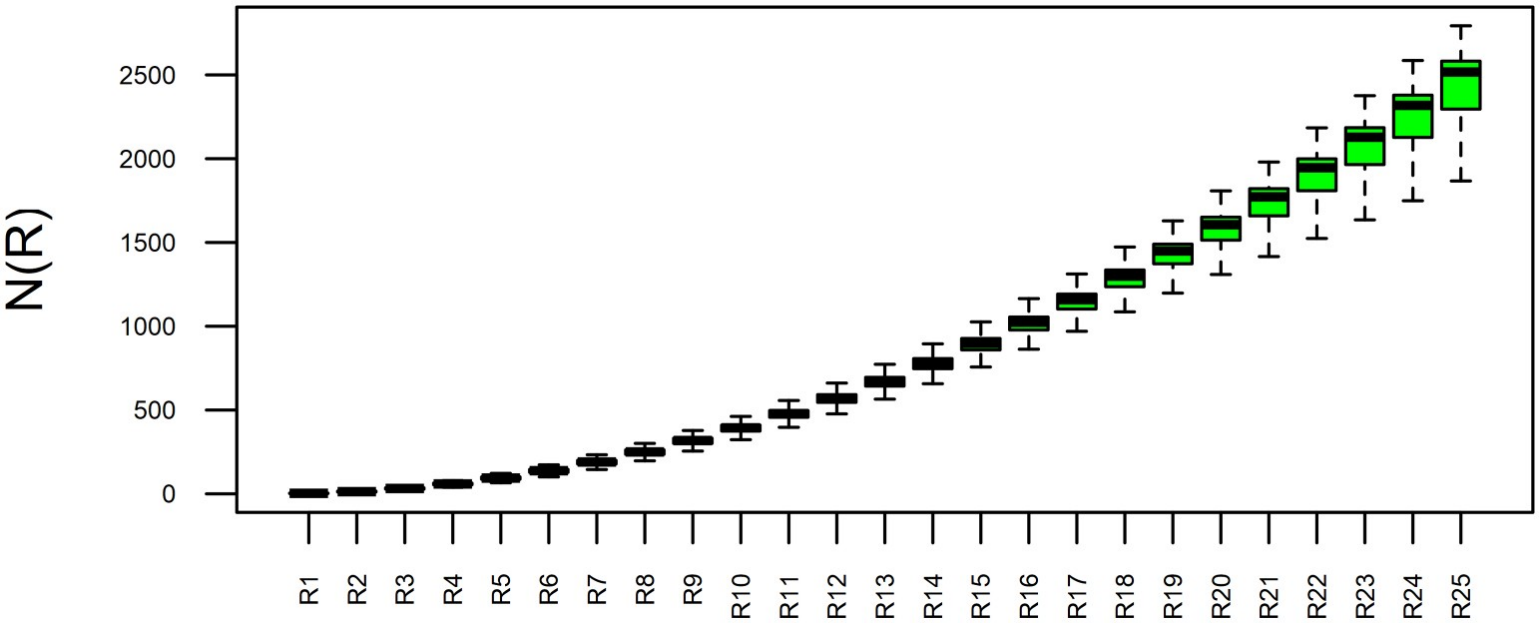
Boxplots of the Swiss CSR local growth curves.

**Fig 8:**
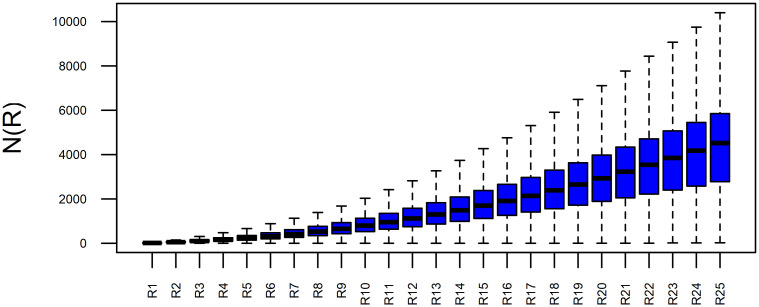
Boxplots of the Swiss population local growth curves.

General overview and the differences between local growth curves for CHCSR (Fig 7) and population data (Fig 8) can be seen in the figures, where the curves are represented using box–plot technique. At each radius *R*_*i*_ box–plots show the level and variability of *N*(*R*_*i*_)—number of inhabited cells within a circle of *R*_*i*_. It is evident, that the variability of LGC for the population data is much higher, especially at the end of the curves (large radii).

**Fig 9:**
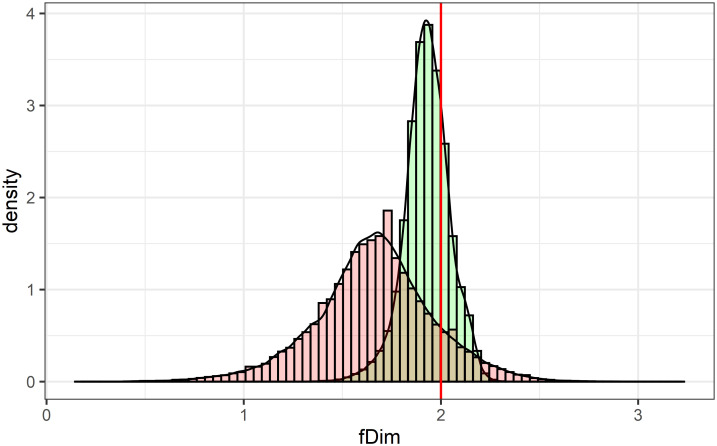
Distributions of the local fractal dimensions for the CHCSR and Swiss population.

The histograms of local fractal dimensions (fDim) for the CHCSR (green) and population (red) data are shown in Fig 9. Both distributions are biased to the left from the reference level of *fd*_*ref*_ = 2, corresponding to the non clustered pattern in a two dimensional space.

The distribution of CSR pattern has less variance and is concentrated near the level of validity domain natural clustering. It is interesting to note, that both are symmetric and close to the Gaussian distributions with the following parameters: *Mean*_*pop*_ = 1.66, *StandarDeviation*_*pop*_ = 0.3, for the population data; and *Mean*_*CHCSR*_ = 1.93, *StandarDeviation*_*CHCSR*_ = 0.11 for the random pattern. The distribution of population local fractality is rather unexpected, taking into account the complexity of Swiss topography and clustering complexity of the pattern.

**Fig 10:**
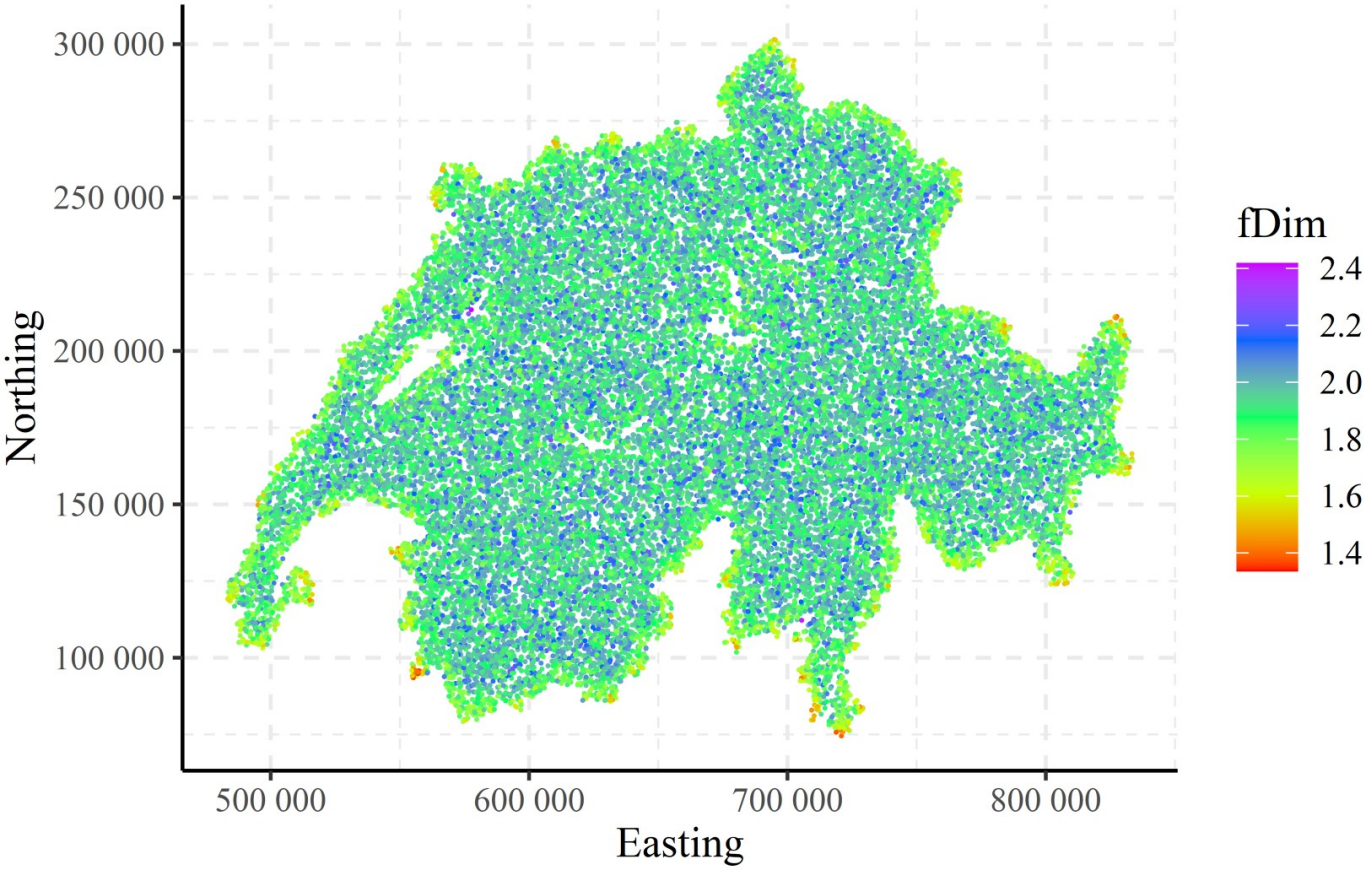
CHCSR: Spatial distribution of the local fractal dimension.

**Fig 11:**
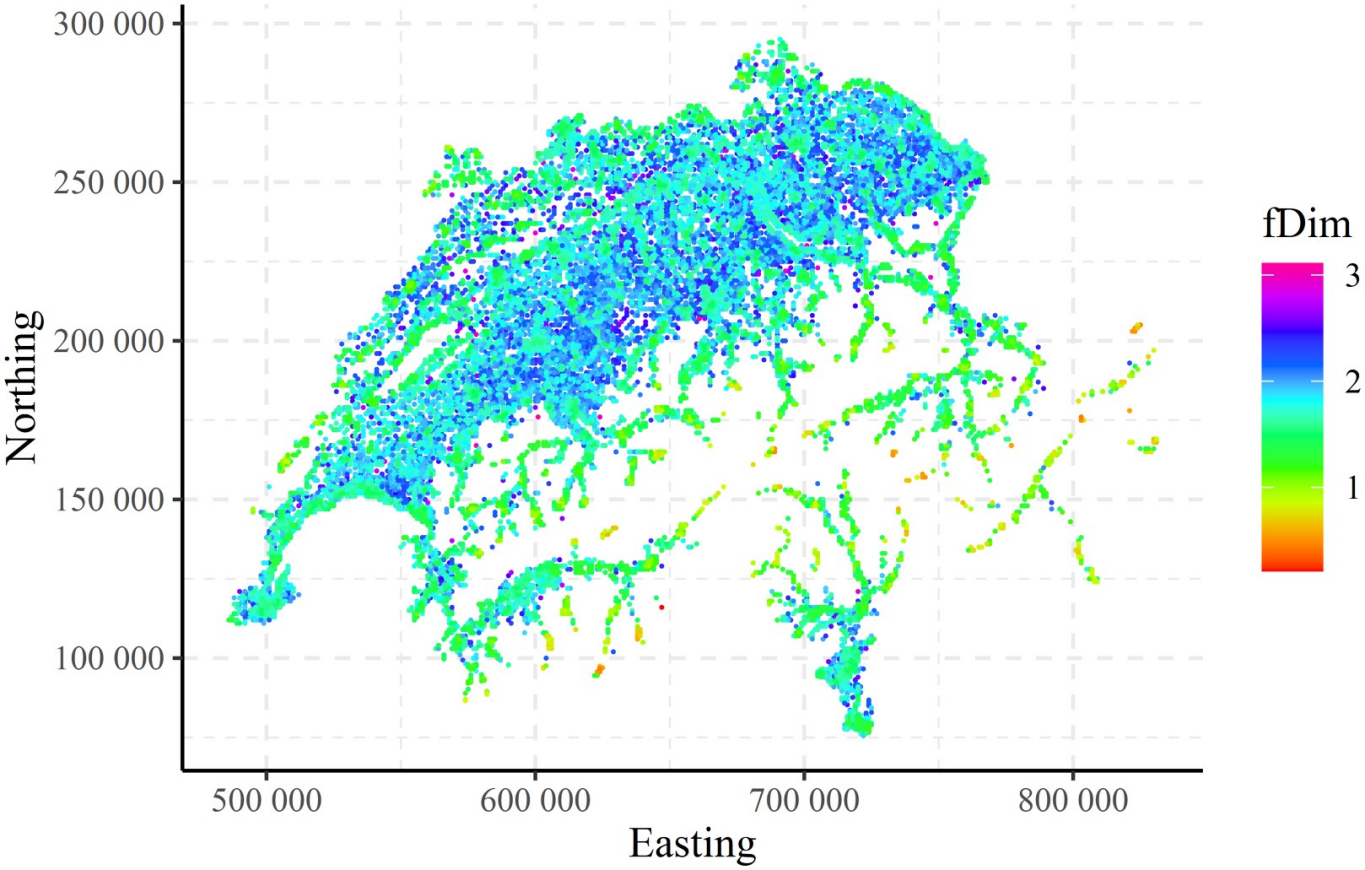
Swiss population: Spatial distribution of the local fractal dimension.

The spatial distributions (maps) of the local fractal dimension (fDim) for the CHCSR and population patterns are shown in Figs 10 and 11. For the visualisation purposes only a part (50000) of the data was randomly selected.

#### Intrinsic dimension estimation

According to the LGC principle of computation, they are positive and monotonically increasing, smooth and convex functions. In the present study they are represented by points in a 25-dimensional space. Therefore, it is reasonable to study their real—intrinsic dimension, defining the degrees of freedom (number of free parameters necessary to parametrised them). When ID is estimated using fractal techniques, it can be a non integer number [33].

There are many approaches and methods used to estimate intrinsic dimension of data. This is a very interesting and fundamental question, especially in high dimensional feature spaces, see, for example, [33–36] and references therein.

In the present research ID is estimated by applying the Morisita index of clustering [35]. The following results were obtained: *ID*_(*CSR*)_ ≃ 3.06 for the CSR pattern and *ID*_(*pop*)_ ≃ 3.11 for the population data. Qualitatively we can interpret these results that the intrinsic dimension of data are between 3 and 4. An independent estimation of IDs based on Reniy’s entropy gives similar results. Thus, the data (LGC) embedded into 25—*dimensional* space in reality have much smaller intrinsic dimension. It means, that data can be considered in a lower dimensional space, without loosing significant information. This result is understandable, if we take into account properties of the LGC mentioned above. Having shapes close to quadratic function, they can be parametrized by three parameters—level, slope and curvature. It opens a way for the parametric study of the LGC.

As it was shown in [31, 35], Morisita index can be efficiently used not only to estimate the intrinsic dimension of data, but also to rank the input variables according to their redundancy. This topic is considered in the following section.

#### Redundancy analysis

Classical way to study the redundancy in data, is to perform a principal component analysis (PCA). PCA is based on correlations and is a linear approach. PCA belongs to the feature extraction algorithms.

**Fig 12:**
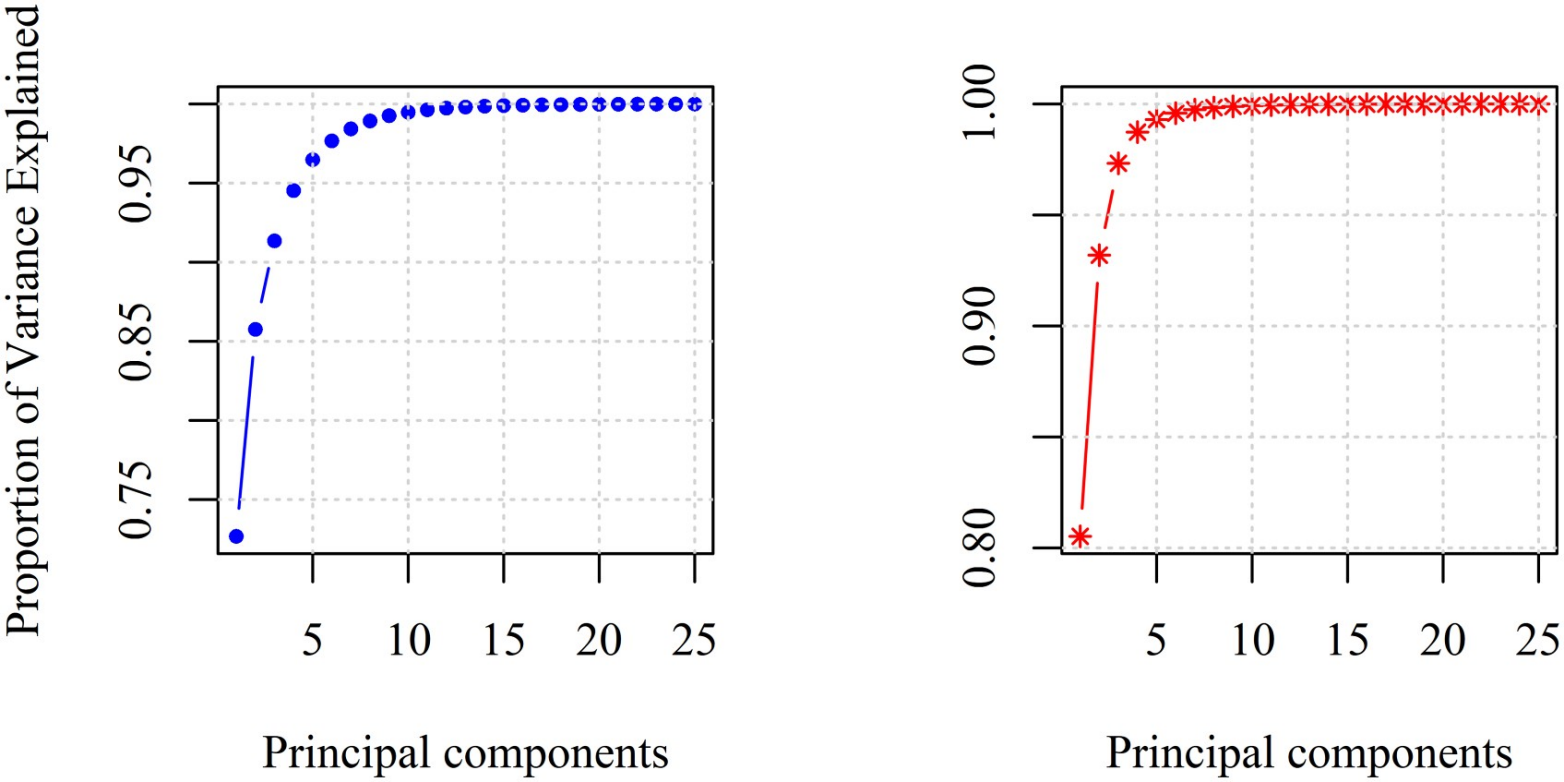
PCA analysis of CHCSR (left) and population data (right).

The variance explained in data by principle components, is shown in Fig 12 for both data sets.

PCA analysis confirms the redundancy demonstrating that only few components from 25 inputs are contributing to explain the variance in data.

**Fig 13:**
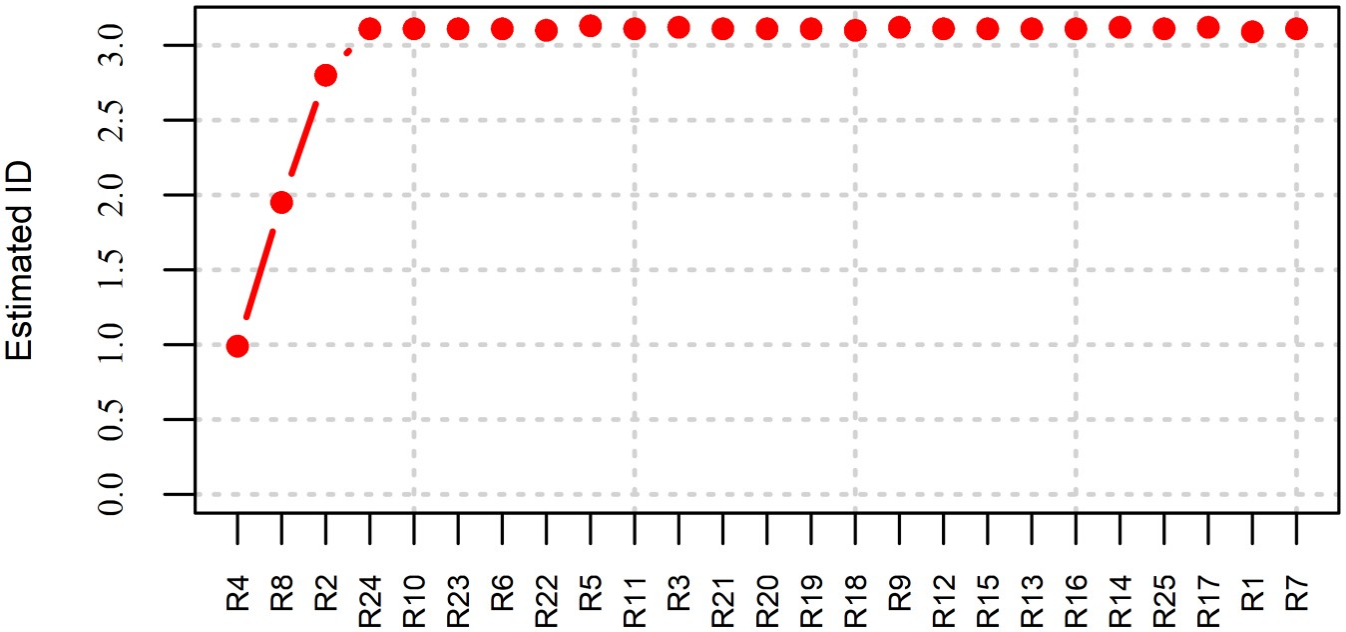
Redundancy analysis in Swiss population LGC.

Non-linear analysis on redundancy in data can be performed using Morisita index [31]. The Morisita based redundancy reduction algorithm ranks the input features according to their contribution to the increase of ID, see Fig 13. The features which do not contribute to the increase of ID are considered as redundant, [31]. This algorithm belongs to the class of feature selection algorithms—selection of pure features without mixing them like in PCA (feature extraction) [31].

The following non-redundant variables were selected by the algorithm based on Morisita ID: *R*4, *R*8, *R*2, *R*24. It is interesting to mention, that selected variables well represent the curve at different scales—local, intermediate and global.

Later, we will study clustering in the complete input space and in the space with removed redundancy.

## Unsupervised learning

Unsupervised learning in this study deals with two related tasks: dimensionality reduction and clustering. The problem is unsupervised, because only the information about input feature space, composed from local growth curves, is considered.

Another type of the analysis can be based on functional data analysis, when curves are considered as functions to be clustered [37]. This topic is beyond the scope of current study.

Clustering is a fundamental task in statistics and machine learning. The problem is ill-defined, therefore an expert’s opinion in the domain plays a crucial role in performing clustering analysis and interpreting the results [38].

In this paper clustering is performed according to the methodology following several important steps:

- Construction of the input feature space. In our case it is a 25-dimensional space composed by LGC.
- Data pre-processing (scaling, transformations, missing values and outliers analysis, etc).
- Dimension reduction (removing the redundancy).
- Clusterability. Answering the question: are data really clustered?
- Determination of the number of clusters.
- Selection and training of the method, clustering.
- Validation and interpretation of the results.
- Visualisation of data, results and models.
- Understanding and interpretation of the results.

When analysing clusterability, or clustering tendency, it is important to take into account the validity domain. Therefore, in the paper a comparison between two data sets—real and simulated, is essential in order to understand and interpret the results.

The decision on the number of clusters in real data case studies is a challenging task [38, 39]. There are many criteria proposed to examine this problem. Often, many of them are considered together to propose a decision [40].

### Clustering

There are very many methods and algorithms to perform clustering of different types of data: classical *k*–means, density-based clustering, model-based clustering, partitioning methods, hierarchical clustering, fuzzy clustering, etc. The literature on clustering is vast and covers many topics and applications. Some introduction to the basic and advanced clustering methods and techniques can be found, for example, in [41–46].

Depending on data quality and quantity, their properties and complexity (dimensionality) etc., the relevant approach should be selected. Normally, several methods are applied to compare the results and their validity and stability.

The following methods were applied in clustering of local growth curves in this paper:

- *k*–means
- *k*–medoids: pam and CLARA (clustering large applications based on *k*–medoids)
- hierarchical clustering
- self-organizing Kohonen maps (som)

The choice of the clustering algorithms was guided by the following considerations: *k*–means was selected as a “benchmark” algorithm, CLARA algorithm has demonstrated extreme computational efficiency and gave good results, hierarchical clustering is both clustering and visualisation tool, and som can be considered as a standard unsupervised algorithm in machine learning.

An advanced study will apply nonlinear techniques like kernel *k*–means, nonlinear/kernel PCA, etc. Spectral clustering—going from data points to graph representation and graph partitioning problem, is an interesting approach to be considered because it does not make any assumption about the shape of clusters. From another side, DBSCAN algorithm does not need a priori knowledge about the number of clusters. More complex and more powerful algorithms, means more difficult to train and interpret the results. These topics lie outside of the scope of the present paper.

It should be noted, that the clustering methods selected were able to detect important and interesting patterns in data.

In the following, first, let us consider the problem of clustering tendency (clusterability) or the question about the presence of clusters in data.

#### Clustering tendency

The clustering tendency was evaluated using different measures considered in [47]. The hypothesis of “no clusters” was rejected both for simulated and population data. Let us remind, that clusterability was analysed in a 25–dimensional LGC space as well as after the redundancy was removed by “IDmining” approach [48].

Visualization of data is a very valuable tool in clustering analysis. Unfortunately, when studying high dimensional and big data sets, visualisation becomes a challenge.

**Fig 14:**
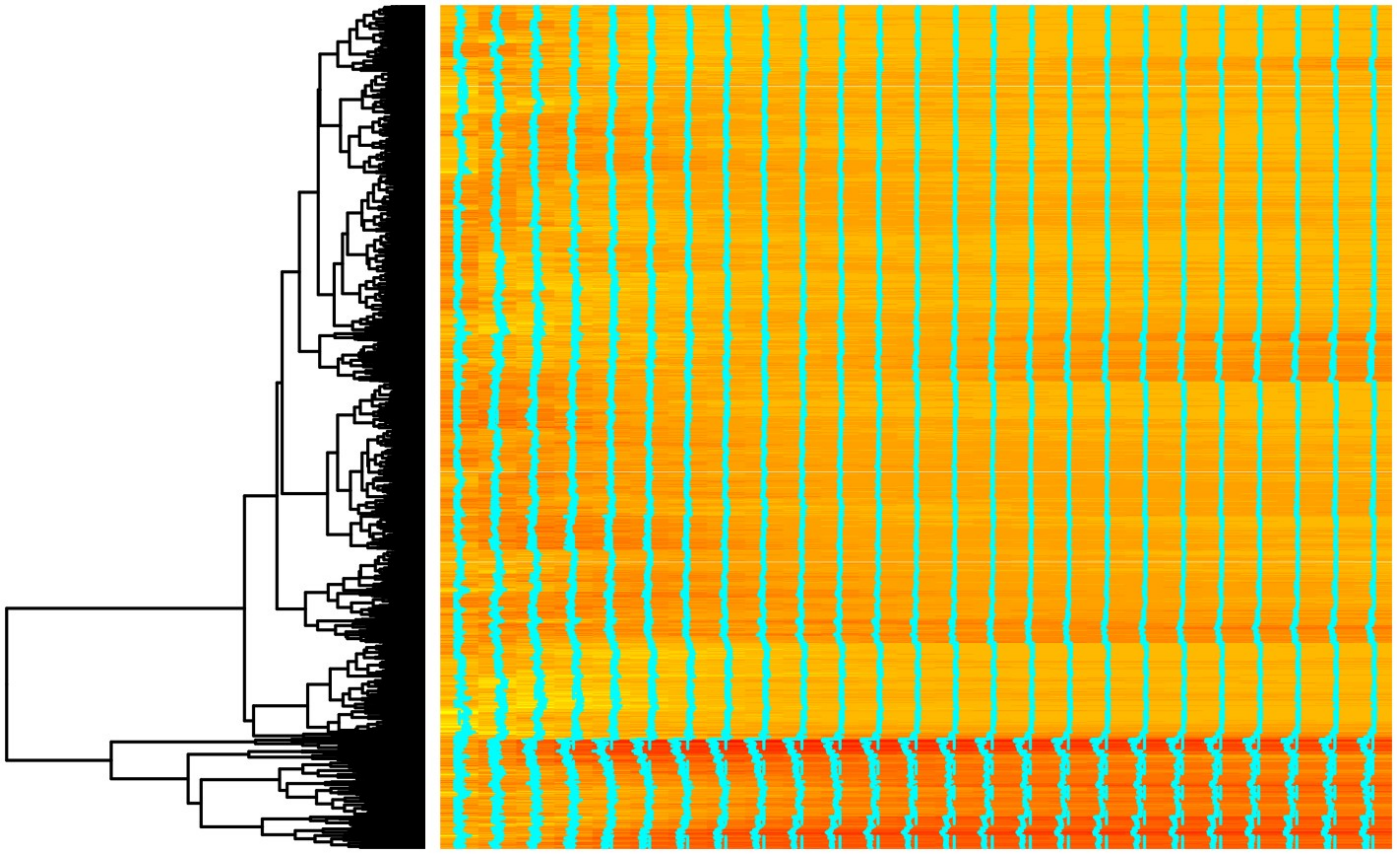
Heatmap presentation of CHCSR data.

**Fig 15:**
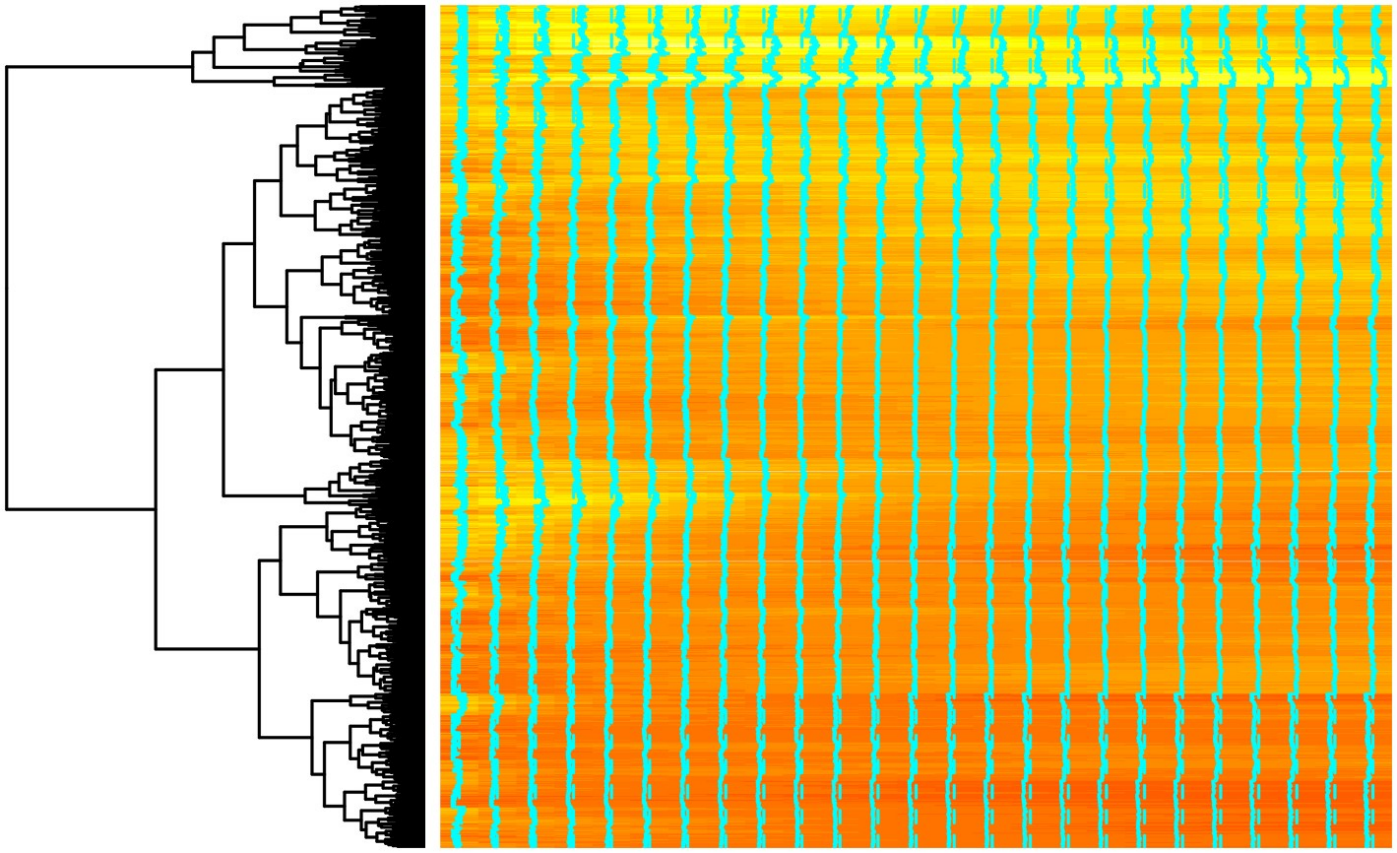
Heatmap presentation of Swiss population data.

Well known technique combining the visualisation and hierarchical clustering—heatmaps, was applied to LGC. For the visualisation purposes, from original data 5000 randomly selected curves were reordered according to their similarity (euclidean distance) and visualised as heatmaps along with dendrograms, see Figs 14 and 15 for CHCSR and Swiss population data, correspondingly. Heatmaps clearly confirm clustering in curves and the number of clusters can be estimated cutting at different height of dendrograms.

#### Number of clusters. Clustering validation

The question of “how many clusters?” is closely connected to the problem of clustering validation. In most cases the clustering validation criteria, often defined as objective functions, are also used to make a decision about the number of clusters.

The literature on clustering validation and definition of the clusters number covers many different aspects at different levels, see, for example, [40, 41, 47, 49].

In most cases clustering validity indexes are considered just as guides, while final decision is being made by an expert [38]. Application of different visualization tools provide an important help in making decision about the clustering validity and the number of clusters [50, 51].

In order to define clustering validity index (CVI), usually two natural concepts are used: compactness (e.g., variability of distances within the same cluster) and separability (the dissimilarity between the clusters, e.g. distance between different clusters, defining how well they are separated). In general it can be represented as

$$\begin{array}{c} CVI=\frac{f_{1}\left(Separability\right)}{f_{2}\left(Compactness\right)} \end{array}$$ (2)

where *f*_1,2_ are the functions, in many cases linear, of the “Separability” and “Compactness”.

Some indexes are constructed using also measures based on densities and even on local fractality, see, for example, [26].

This problem in the present paper is solved using two approaches: 1) following the NBclust methodology, i.e., fixing a clustering method and computing 30 indexes of clustering validation by changing the number of clusters [40]. Each index selects the best number of clusters and final number is a majority vote. 2) Using visualization of hierarchical clustering via dendrograms. It gives qualitative and quantitative impression on data similarity and clustering. In the latter case only a randomly selected part of LGC is used.

In the former case the algorithm selected can influence the decision. Therefore, several clustering methods should be applied and expert knowledge is important. For the scientific reason, different number of clusters around the optimum one can be considered.

The NBClust approach voted for two clusters in CHCSR data and between two and up to six clusters for the population data, depending on the clustering algorithms used. The most frequent number was three. Therefore, in the following we will concentrate on 2 clusters for CHCSR data set and between 2 and 4 clusters for the population data set.

#### Clustering results

Using the information about the optimal number of clusters proposed by several criteria, the clustering algorithms can be applied.

*K*–means can be considered as a standard/benchmark algorithm. It is simple and intuitively clear method widely applied in clustering tasks [41, 43, 44].

More robust and stable method, replacing *k*–means, is the class of *k*–medoids [43]. *K*–medoids is similar to *k*–means, but is based on data, i.e. centers of the clusters are real data points. In short, it selects *k* data points as clustering centers, which minimize the dissimilarity in the cluster. These data points are called medoids and the general algorithm is called PAM—partitioning around medoids.

In order to work with large data sets, which is our case, the pam algorithm was modified to develop a CLARA (Clustering for Large Applications) algorithm [43]. The CLARA algorithm is performing *k*-medoids search on a subset of data, therefore it is adapted to large data sets. It is fast and efficient algorithm for clustering tasks, having also advantages of *k*-medoids. Both algorithms are implemented in many public software libraries.

In the present paper the results of *k*–means, which are qualitatively similar to *k*-medoids, are not shown.

**Fig 16:**
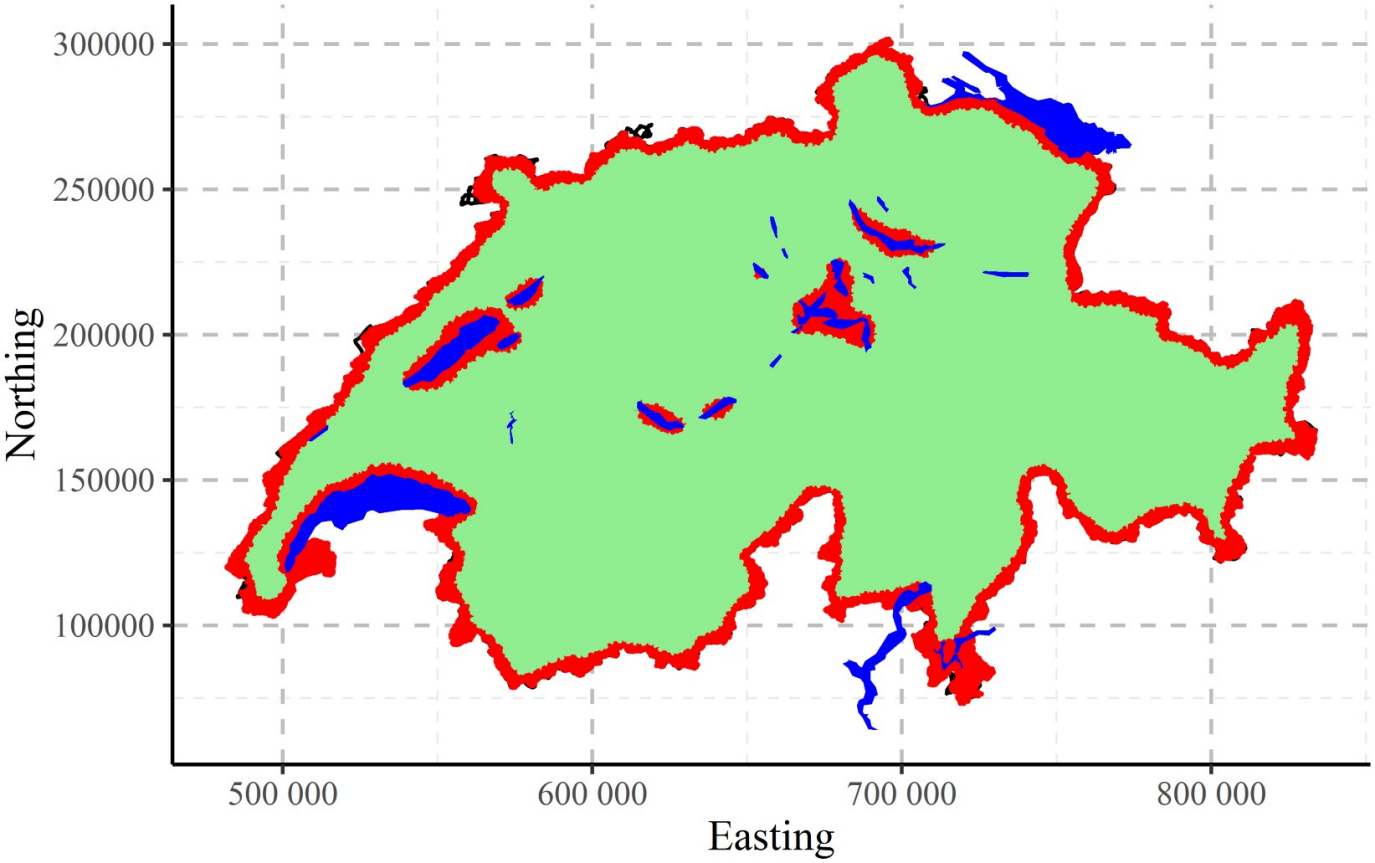
CLARA clustering of CHCSR data into two clusters.

The map of clustering CHCSR data performed by CLARA algorithm into two clusters is presented in Fig 16. The result is very clear: one cluster corresponds to the border of the country and also recognizes the coasts of lakes.

**Fig 17:**
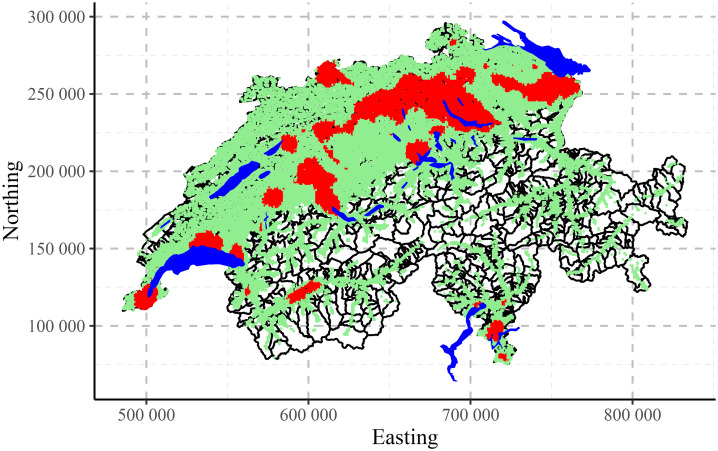
CLARA clustering of Swiss population data into two clusters.

**Fig 18:**
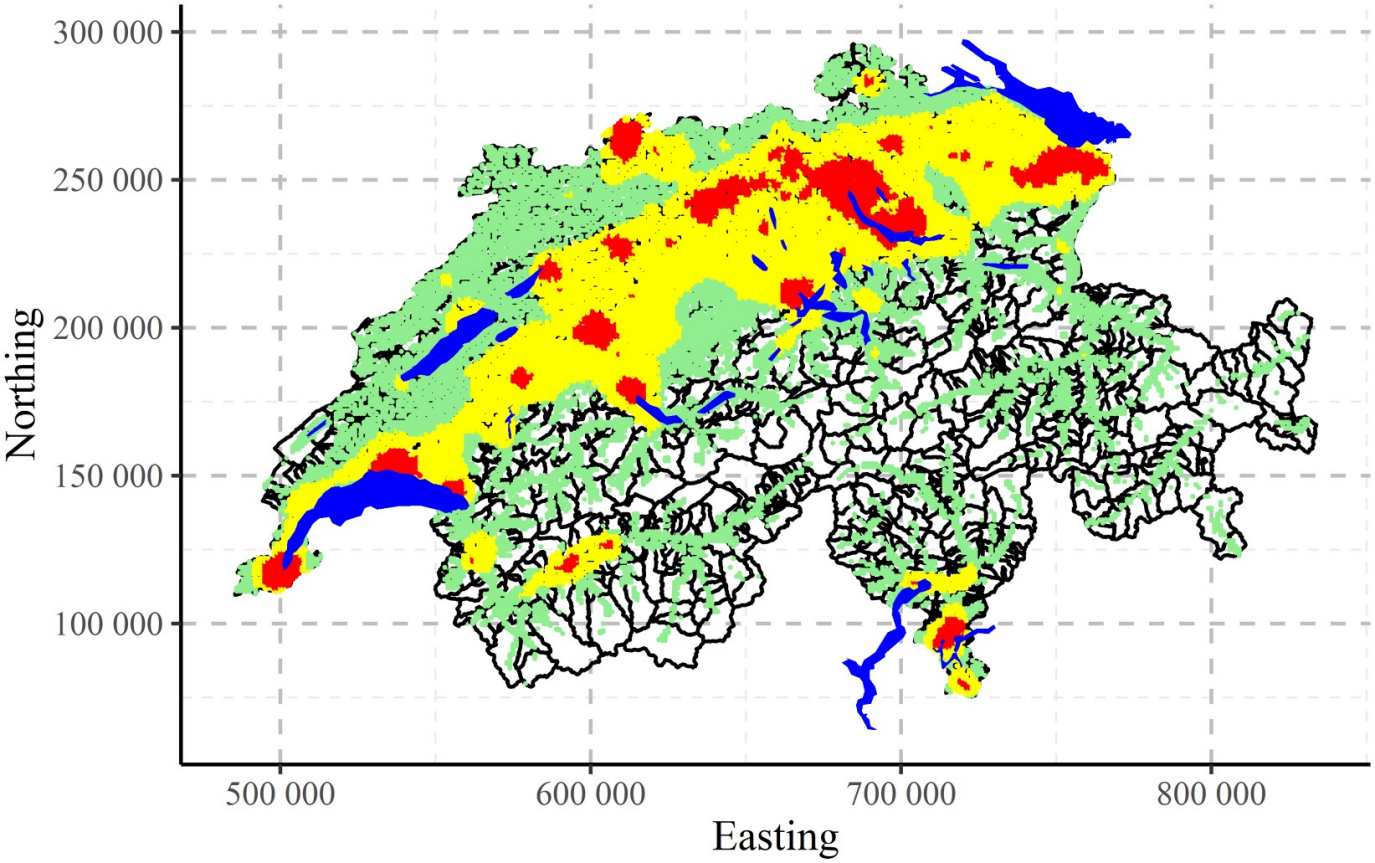
CLARA clustering of Swiss population data into three clusters.

**Fig 19:**
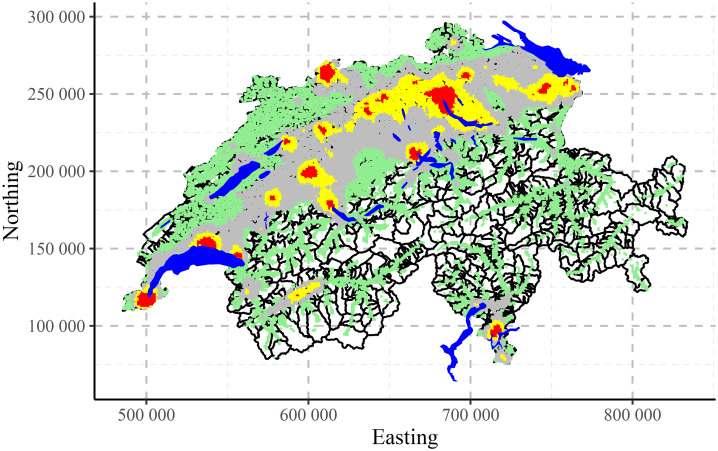
CLARA clustering of Swiss population data into four clusters.

The maps of clustering for Swiss population data performed by CLARA algorithm are shown in Fig 17 for two, Fig 18 for three and Fig 19 for four clusters, correspondingly. Clustering algorithm has recognised populated agglomerations. Adding more clusters, produces more details in the patterns until “overfitting” is observed.

**Fig 20:**
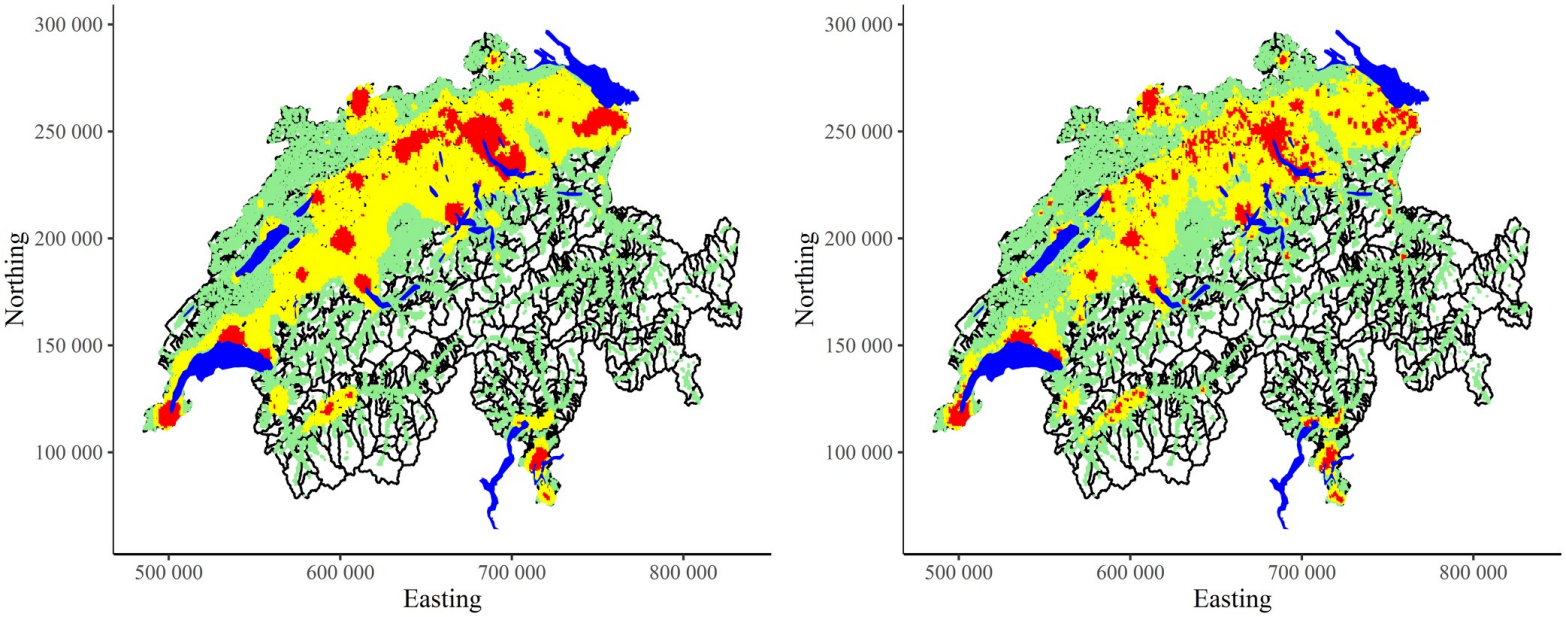
CLARA clustering of Swiss population data into three clusters: Complete input space (left) and removed redundancies in input space (right).

The comparison between two CLARA clustering results—with all variables (left, 25-dimensional input space) and with removed redundancies (right, 4-dimensional input space)—are shown in Fig 20). Qualitatively the results are very similar. Quantitatively, in the reduced space there is more variability and some redistribution between clusters. Therefore, by keeping only 4 non-redundant variables, it was possible to reconstruct almost the same clustering structures.

### Self-organizing maps

Self-organizing (Kohonen) maps (SOM) is a well known and widely used approach in unsupervised machine learning [52, 53]. SOM have many interesting properties useful in high dimensional data dimensionality reduction and visualization. SOM are also popular in Geographical Information Sciences [54, 55].

Let us briefly recall the basic principles of SOM.

#### SOM principles

Self-Organising Map (SOM) is a single layer feedforward network with 2D (usually) grid of ordered neurons. It means, that each neuron has its own 2D coordinates (row/column) on the map and is associated to data with a weight vector.

After the network was properly initialized (randomly in this research), there are the following processes in the formation (unsupervised learning) of the **self-organizing map** [52, 56]:

- Competition
- Cooperation
- Adaptation

These processes give rise to the global ordering of neurons (nodes of the grid)—self-organisation of the network and reflection of the topological structure of data: more similar data are associated with nodes that are closer in the grid, whereas less similar are situated gradually further away in the grid [53].

In the present study several packages in R (“SOMbrero”, “kohonen”, “popsom”) and “GeoSOM” module from [54] were used to carry out the SOM analysis of data.

#### SOM results

After training, data from high dimensional space are topologically projected onto a two-dimensional grid, according to the SOM rules. U-matrix is one of the most used visualisation tool along with the slices—SOM layers corresponding to the input variables. There are as many layers as input features. Next step is to cluster the SOM-map. This step can be done using any clustering method. The most used are hierarchical and *k*-means methods.

In the following a software module “GeoSOM” from “Machine Learning Office” was adapted [54] and the main results are shown. The basis of “GeoSOM” follows the classical version of Kohonen’s SOM [52]. The SOM with empirically selected hexagonal grid [20x15] was trained both on CHCSR and Swiss population data.

The results of SOM modelling are in qualitative and quantitative agreement with other clustering methods presented above, which means that clustering is stable across the algorithms.

**Fig 21:**
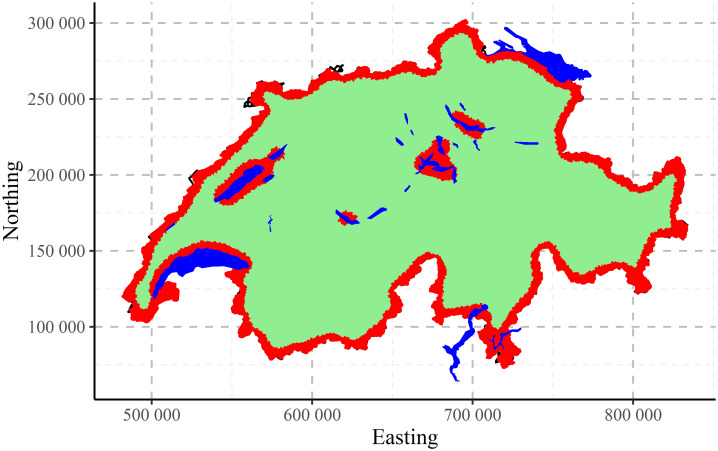
SOM clustering of local growth curves. CHCSR data, two clusters.

**Fig 22:**
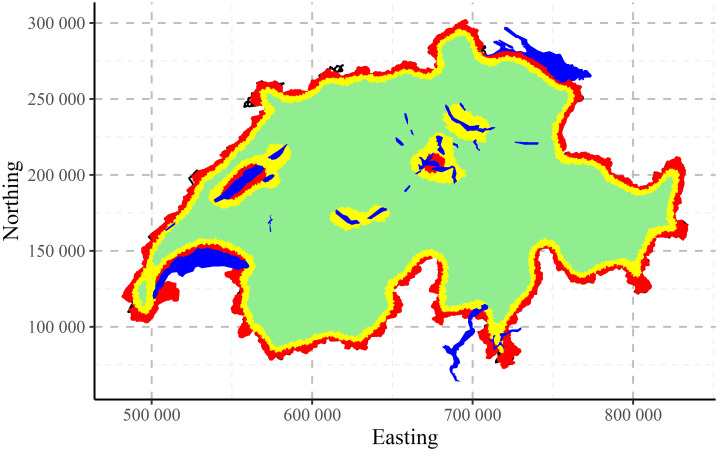
SOM clustering of local growth curves. CHCSR data, three clusters.

SOM CHCSR data clustering into 2 and 3 clusters are shown in Figs 21 and 22. It is interesting to note, that if we add the third cluster, it marks the intermediate zone between boundary and main cluster.

**Fig 23:**
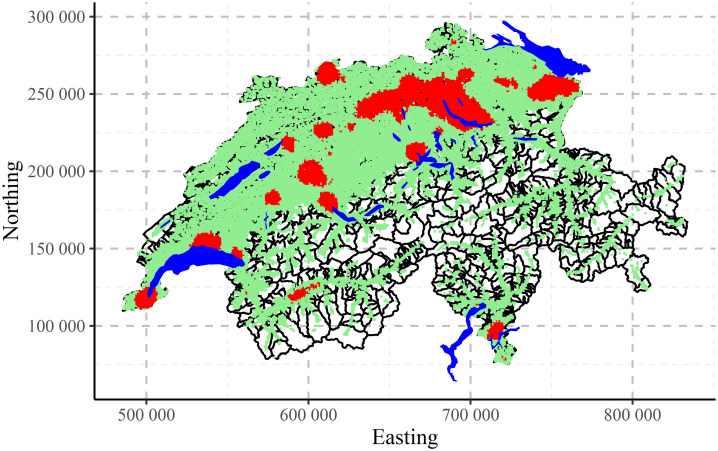
SOM clustering of local growth curves. Swiss population data, two clusters.

**Fig 24:**
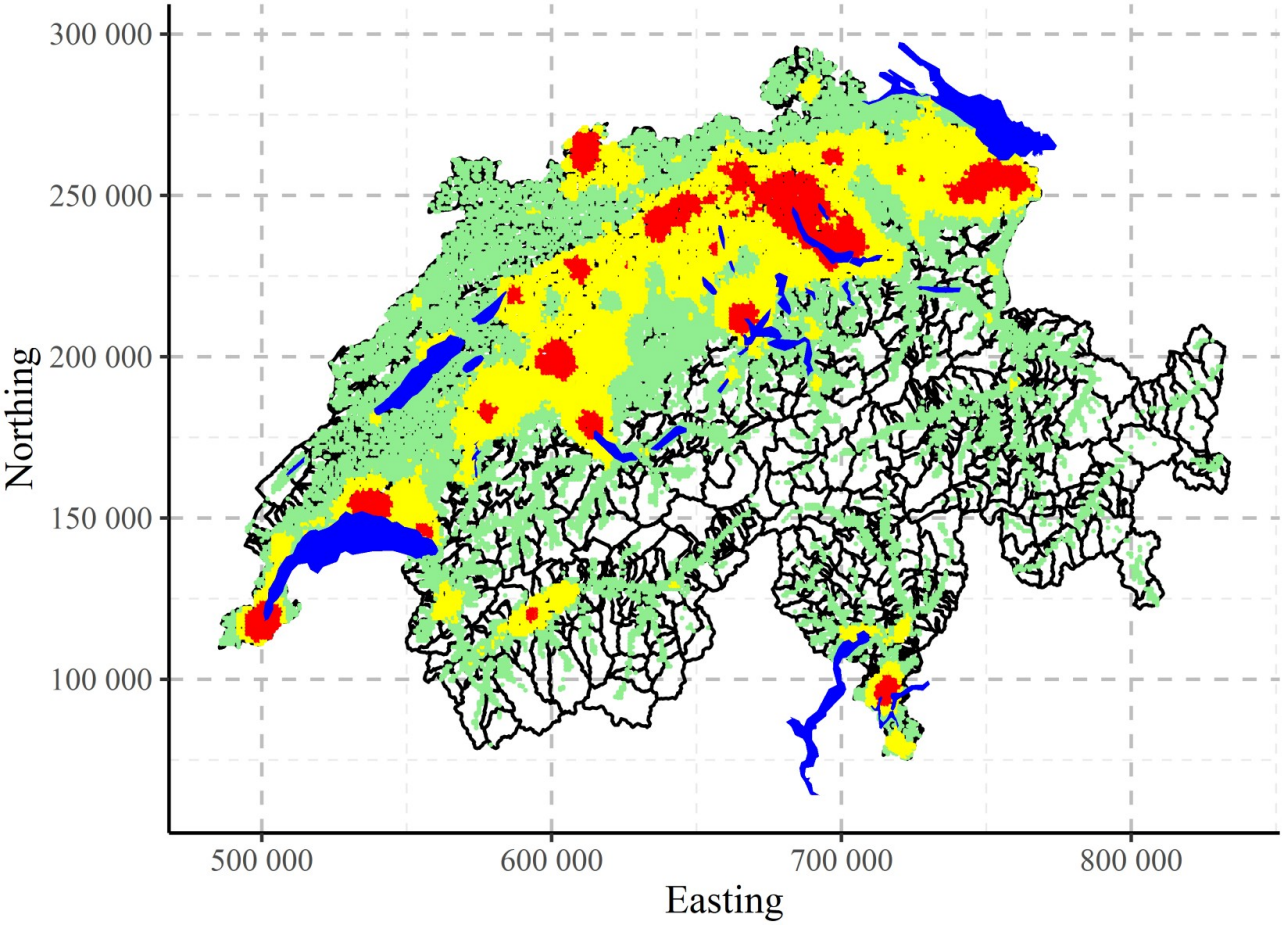
SOM clustering of local growth curves. Swiss population data, three clusters.

The SOM population data clustering are shown in Figs 23 and 24

### Understanding of clustering

In order to better understand and interpret the results obtained by clustering algorithms, let us have a look at the distributions of local fractal dimension (fDim) and the number of cells within the fixed radius, which characterises the local density for each cluster. It will help us to look at the results taking into account two important characteristics of data: local dimensionality and local density estimated by counting the number of points within a given radius (*R* ∼ 4000*m*).

**Fig 25:**
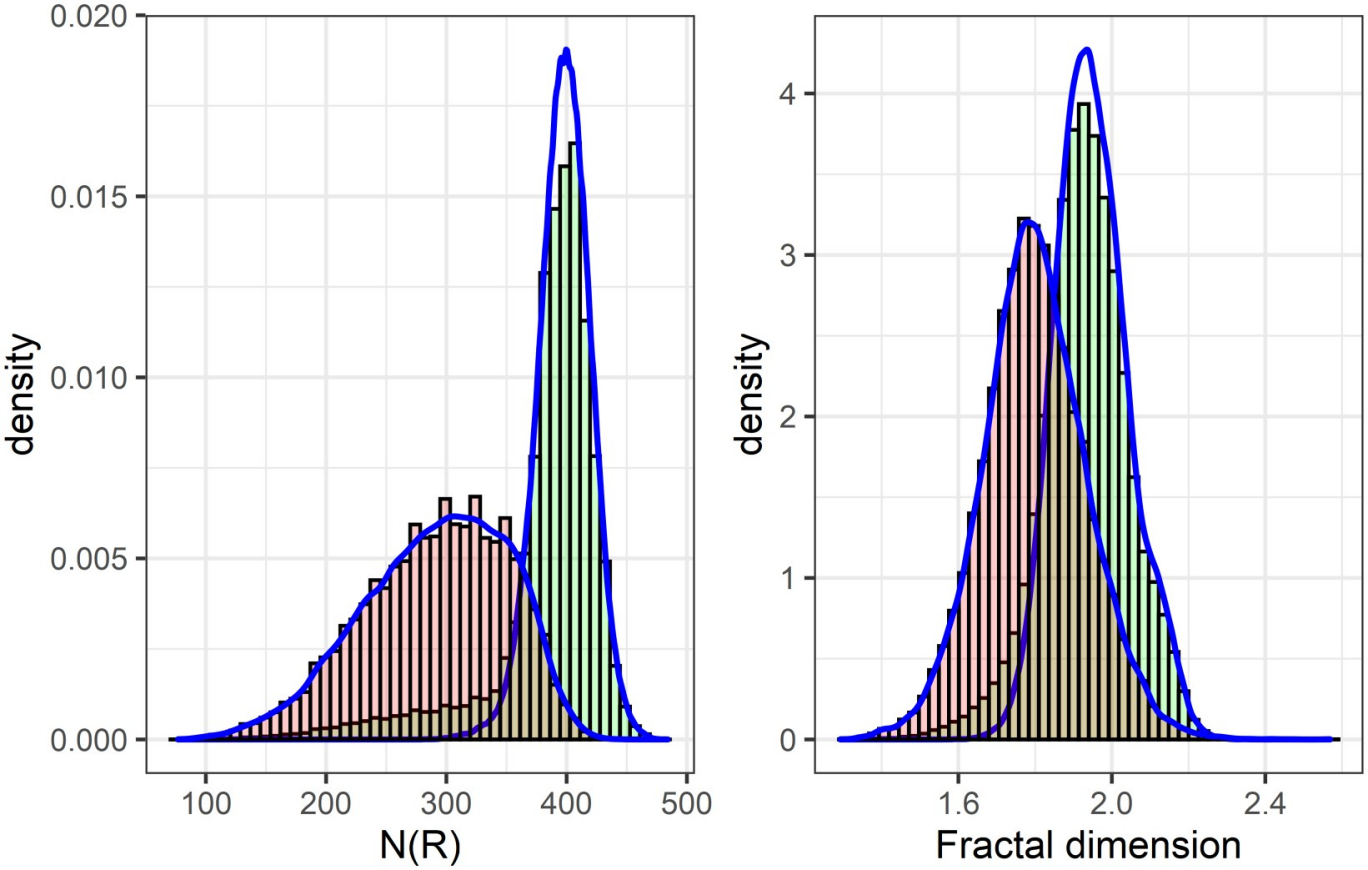
Distribution of the number of points within the radius *R* ≃ 4000 m for two clusters (left). Distribution of the local fractal dimension for two clusters (right).

The histograms for local fractal dimension and local density after CLARA clustering of CHCSR data are presented in Fig 25.

In this case both factors (density and dimensionality) are clearly contributing to the clustering decision.

**Fig 26:**
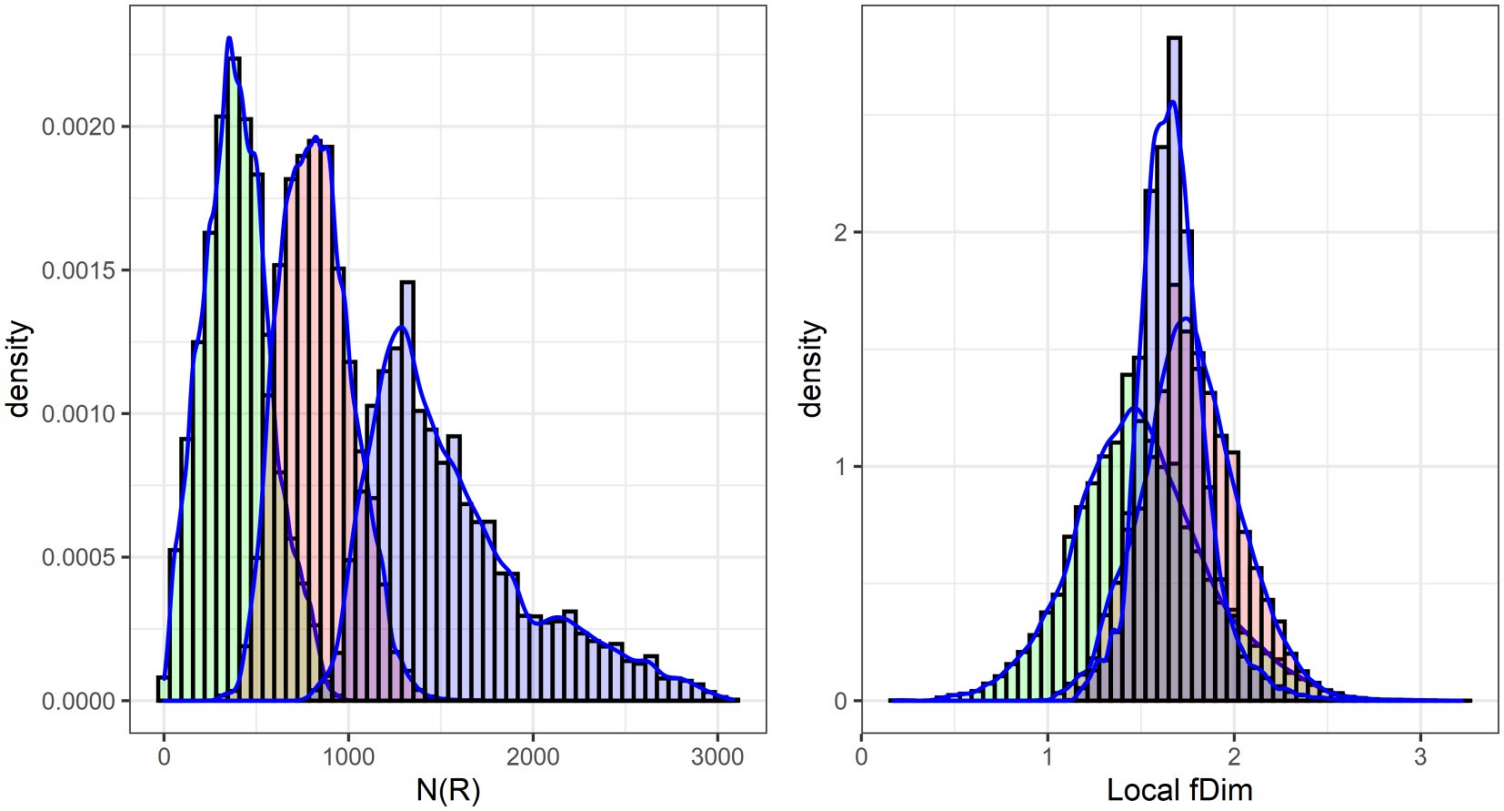
CLARA clustering of Swiss population data into three clusters: The distribution of local density (left) and the distribution of local fractal dimension (right).

The same analysis was carried out for Swiss population data, see Fig 26.

For the population data it is the density which dominates the partitioning into clusters.

## Discussion and conclusions

In the present research spatial patterns of Swiss population distribution using fractal concepts and unsupervised learning algorithms are studied. High resolution data (inhabited [100*m*×100*m*] cells), considered as a point process were embedded into a 25-dimensional space via local growth curves, which were computed from 300 to 10000 meters distances. The feature space constructed is used for the unsupervised learning (clustering) of Swiss population distribution.

Let us note, that the input feature space applied in this paper is only one from many other possibilities, and it takes into account basically only geometrical aspects of the population distribution. Nevertheless, it is an interesting approach, because it considers both density of inhabited cells at different spatial scales as well as the dimensionality, i.e. local fractal dimension. As a reference data set a random pattern (CHCSR) was generated within the validity domain and compared with raw data.

The distribution of the local fractal dimension for Swiss population data is quite symmetric and has three times larger standard deviation than the random pattern. Of course, spatial distributions of real and simulated data are very different.

Comprehensive exploratory analysis of the LGC confirms that data are clustered in the input feature space. Using different criteria and measures, it was estimated, that the random pattern can be optimally described by two clusters. These clusters are well separated and have a simple and clear interpretation: one is concentrated on the boundaries and the second one occupies internal region of the county.

On the contrary, according to different clustering algorithms and validation criteria, the characterisation of real population data demands several clusters: from three to six which characterizes with different details population agglomerations. More clusters contribute with details of already observed patterns, which, finally, gives rise to “overfitting”.

The future research can be foreseen in several directions:

1. practical improvements: application of nearest neighbours and maximum likelihood approaches in estimating local fractality
2. methodological and algorithmic improvements: multifractal analysis, functional data analysis of LGC, multivariate extension of the approach, consideration of the number of inhabitants per cell
3. Input space feature engineering with a variety of feature selection and feature extraction algorithms
4. application of advanced unsupervised learning approaches: kernel k-means, spectral clustering, nonlinear PCA, dbscan, etc. These algorithms will help to detail the complexity/nonlinearity of the phenomena and reinforce the methodology
5. adaptation to other countries and regions, more comprehensive and complete studies
6. application of unsupervised learning of local fractality (local ID of data manifold) to other important case studies: environmental risks, natural hazards, etc

## Supporting information

S1 Data (ZIP)
